# Fabrication and *In-vitro *Evaluation of Buccal Mucoadhesive Tablet of Meloxicam 

**DOI:** 10.22037/ijpr.2019.111820.13378

**Published:** 2020

**Authors:** Mahshid Arabi, Seyed Alireza Mortazavi, Zahra Jafariazar, Hassan Farhadnejad, Golnoosh Alipour Harisa, Yousef Fatahi

**Affiliations:** a *Department of Pharmaceutics, Faculty of Pharmacy, Islamic Azad University, Tehran, Iran. *; b *Department of Pharmaceutics and Pharmaceutical Nanotechnology, School of Pharmacy, Shahid Beheshti University of Medical Sciences, Tehran, Iran. *; c *Student Research Committee, Department of Pharmaceutics and Pharmaceutical Nanotechnology, School of Pharmacy Shahid Beheshti University of Medical Sciences, Tehran, Iran. *; d *Department of Pharmaceutical Nanotechnology, Faculty of Pharmacy, Tehran University of Medical Sciences, Tehran, Iran. *; e *Nanotechnology Research Center, Faculty of Pharmacy, Tehran University of Medical Sciences, Tehran, Iran.*

**Keywords:** Meloxicam, Mucoadhesive, Buccal tablet, Drug delivery, HPMC, Carbopol

## Abstract

In this study, buccal mucoadhesive tablets of meloxicam were formulated for drug delivery as an alternative route. Direct compression method was applied for the preparation of tablets. Also, different polymers, including hydroxypropyl methyl cellulose (HPMC) 1000, 4000, and 10000, as well as carbopol 934p and carbopol 971p were used as the mucoadhesive polymer and retardant polymer. Thirteen formulations were investigated with various concentrations of polymers. The physicochemical characteristics, *in-vitro *drug release, swelling index, and taste modification of tablets were evaluated. Also, Carr’s index and Hausner ratio were studied. In addition, zero-order, first-order, and Higuchi kinetics were investigated and the results showed that the highest correlation coefficient (R^2^) is related to zero-order kinetic for formulations B_2_ and B_3_. Furthermore, the highest R^2^ is related to Higuchi kinetic for formulation C_3_. Formulation B_2_ showed the maximum release of 99% in 12 h. The results demonstrated that Formulation B_2_ can be considered as a proper buccal mucoadhesive tablet of meloxicam with desired property.

## Introduction

Oral route is known as the most appropriate route of administration for drug delivery among all other routes that have been explored (1). It is the most favorable route due to its ease of administration, high patient compliance, cost-effectiveness, and flexibility in the design of dosage form (2). However, this site is associated with limitations that restrict its use such as wide first pass metabolism, degradation of drugs in the gastrointestinal tract (GIT), and poor bioavailability (3). Hence, buccal routes can be used as drug delivery because of various advantages against the conventional oral route. These benefits include increasing the bioavailability of the drug while the first pass metabolism is avoided as well as the elimination of presystemic elimination within the GIT. Thus, the oral mucosal cavity is known as a very attractive and suitable site for systemic drug delivery (4). Oral mucosal drug delivery is an alternative site for systemic drug delivery that is easy to administer and well accepted by the patient (5, 6). Regarding the mentioned advantages, mucosal drug delivery has received a great deal of attention, over the past few decades. It can be designed in order to have prolonged retention at the site of application, providing a controlled rate of drug release for improving therapeutic outcome. A new system of drug delivery named mucoadhesive drug delivery system has attracted the interest of pharmaceutical scientists (7). The intestines are enzymatic/alkaline and most of the drugs are destroyed in these environments and unstable in the acidic environment of the stomach. So, the buccal delivery system is the best option for drugs which exhibit low bioavailability, to be compensated by the avoided first pass metabolism. Mucoadhesion is a mechanism that attaches natural or synthetic polymers to a mucosal surface. Physicochemical properties of the polymer, specifications of the biological material, and contact time of the dosage form affect on mucoadhesion stability changes (8). Different buccal mucoadhesive dosage forms have been formulated such as tablets, gels, films, patches, and disks (9-13). The main advantages of buccal tablets include sustained release profile, high permanence time in the buccal mucosa, high bioavailability, and low potential adverse effects. More specifically, buccal tablets are preferred rather than buccal films due to its advantages which include modified release, ability to release drugs at a zero-order rate and maintenance of plasma concentration in therapeutic window for a long time period. In other words, the greatest advantage of buccal tablets in comparison to buccal films is that buccal tablets release the active substance in a sustained release manner of 12 h but buccal films are one of the immediate release forms with 3 h drug release profile. Moreover, other advantages are lower manufacturing cost and larger scale production as compare to other solid dosage forms. So far, different types of mucoadhesive tablet formulations have been described in the literature (14). For example, B. Çelik fabricated and optimized risperidone (RIS) mucoadhesive buccal tablets by direct compression method. They concluded that buccal mucoadhesive tablets show proper physical properties and mucoadhesive strength, and it can be used as an effective formulation for the treatment of schizophrenia (8). P. Mura *et al.* fabricated Polymeric mucoadhesive tablets for topical or systemic buccal delivery of clonazepam by direct compression of combinations of different polymers. The results obtained from their work showed that Carbopol971P/hydroxypropylmethyl cellulose and Poloxamer/chitosan mixtures are the best formulations (15).

Non-steroidal anti-inflammatory drugs (NSAIDs) are a class of drugs that are widely applied to alleviation of pain, fever and inflammation by virtue of the ability to inhibit cyclooxygenase (COX) enzymes which plays a role in biosynthesis of prostaglandins (16). NSAIDs are widely utilized in management of symptoms of osteoarthritis and rheumatoid arthritis (17). Meloxicam is one of the NSAIDs that selectively inhibits the COX-2 activity. Adverse effects of this drug are less than other NSAIDs (18). Thus, the main aim of this study was the fabrication of buccal mucoadhesive tablet of meloxicam and evaluation of its physicochemical properties. In the present study, different polymers including hydroxypropyl methyl cellulose (HPMC) 1000, 4000, and 10000 mPa.s, Carbopol 934 and 971 were selected for various formulations.

## Experimental

*Materials*

Meloxicam powder (Jalinous Pharmaceutical Co., Tehran, Iran), Magnesium Stearate (Vegetable grade, Merck, German), Lactose monohydrate (Ph Eur,BP,NF,JP grade, Merck, German), Microcrystalline cellulose (Avicel® pH 101, Hefei Prote Chemical Co., China), Colloidal silicon dioxide (Ph Eur,BP,NF,JP grade, Merck, German), Hydroxypropyl methyl cellulose (HPMC 1000, 4000 and 10000 mPa.s, ShinEtsu Chemical Co., Japan), Carbopol 934 and 971 (BFGoodrich Co., USA), Ethanol (C_2_H_5_OH, Merck, German), potassium dihydrogen phosphate (KH_2_PO_4_, Merck, German), Sodium hydroxide (NaOH, Merck, German) were purchased from Merck and used as received without further purification. Double distilled water was used throughout the present study.

*Pre-formulation studies on meloxicam powder*

Studies include the analysis of the FTIR spectrum, evaluation of the dispersion and compressibility of meloxicam powder, determination of Carr’s index and Hausner ratio, the determination of the maximum wavelength (λ_max_) using UV spectrum and the construction of calibration curve in the phosphate buffer medium (pH 6.8) to determine the concentration of meloxicam in the release medium. 

*UV spectrum of meloxicam powder*

To obtain the λ_max_ of meloxicam, its stock solution (100 ) was prepared by dissolving 10 mg of meloxicam in 100 mL phosphate buffer (pH 6.8). Afterward, 5 solution was prepared from the stock solution and its UV spectrum was recorded using a Shimadzu UV-Vis spectrophotometer (UV-1650, Japan) in the range of 200-400 nm.

For the construction of meloxicam calibration curve in the phosphate buffer medium (pH 6.8), standard solutions with concentrations of 2, 4, 6, 8, 10, 12, 14, 16, 18, and 20 were made from the stock solution. Their absorbance was recorded at 362 nm. Ultimately, the absorbance of standard solutions versus their concentration was plotted and its equation was obtained.

*Determination of Carr’s index and Hausner ratio*

The Carr index is used as an indication of the flowability of a powder. A portion of the powder was poured into a graduated cylinder up to 10 mL volume (without knocking) and its volume was recorded. Then the graduated cylinder was hit 100 times to the hard surface from 2.5 cm height to make the powder denser. In this case, the volume was calculated again. Then, Carr’s index was computed by Equation 1:

Carr’s index = (p_t _- p_b_/p_t_) × 100 

 (Equation 1)

Where p_t _and p_b_ are tapped bulk density and freely settled bulk density of the powder respectively. Also, the Hausner ratio is related to the flowability of a powder or granular material. It can be introduced as follows (19):

 Hausner ratio = p_t_/p_b _

 (Equation 2)

*Evaluation of the compressibility of meloxicam powder*

One-hundred miligram of meloxicam was filled in the single punch machine and the hardness of the device was adjusted to the maximum extent.

*Formulation of meloxicam tablet*

In this study, five different types of polymer with different percentages were used in the formulation of meloxicam tablet. The fabricated tablets were classified into 5 groups based on the type of polymer in order to facilitate the comparison of the effects of different concentrations of polymers on the characteristics of the pharmaceutical formulation. For the preparation of buccal mucoadhesive tablets, the final weight of each tablet and the amount of pure meloxicam were 100 and 15 mg, respectively. Polymers include HPMC, and carbomer 934 and 971. In addition, Avicel PH 101 was used as the filling material in the formulation. To make any series of buccal mucoadhesive tablets, the powder of drug, filler, and polymer were mixed together. Then, the lubricant was added and tablets with a weight of 100 mg and a hardness of 6-8 kgf were prepared by direct compression method using a press machine with a mandrel of 7 mm diameter. Then, control experiments such as investigating appearance features, thickness, friability, and water absorption were performed on the tablets to select the best formulation.

*Formulations prepared from single-polymer*

Various formulations were prepared using the HPMC 1000, 4000, 10000 polymer, in which constant amounts of meloxicam (15 mg per tablet), magnesium stearate (2% w/w), Avicel (12 mg per tablet) and Aerosil (1 mg per tablet) were used, and the variable factors in these formulations were the amount of the HPMC (10, 20 mg) and the lactose filler. By increasing one of the two factors, the other one was decreased in a way that tablet weight remains constant. The components of these formulations (A_1_–A_6_) are shown in Table 1. On the other hand, the formulations were prepared using carbopol 971, 934 polymer. In these formulations, Amounts of meloxicam (15 mg per tablet), magnesium stearate (2% w/w), Avicel (12 mg per tablet) and Aerosil (1 mg per tablet) were constant. Furthermore, the variable factors were the amount of the carbopol polymer (10, 20 mg) and the lactose filler. When one of these factors increases, the other one reduces as long as the weight of the tablet keeps constant. The components of these formulations (B_1_–B_4_) are shown in Table 2.

*Formulations prepared from two polymers (cellulose derivatives)*

Meloxicam (15 mg per tablet), magnesium stearate (2% w/w), Avicel (12 mg per tablet) and Aerosil (1 mg per tablet) were used in a constant amount per each formulation. Also, the amount of the buccal mucoadhesive polymer (10, 20 mg) and the lactose filler were indicated as the variable factors. By increasing one of these factors, the other one decreases in order to the weight of the tablet kept constant. The components of these formulations are represented in Table 3.

*Evaluation of physicochemical parameters*

*Appearance features*

For the evaluation of appearance features, ten tablets were randomly selected from a series. Appearance features such as color, smell, and health of tablets appearance were considered. All tablets should be uniform and free from any defection and if there is crack, color change, and so on, the tablets will be rejected. 

*Hardness, thickness and disintegration time*

For measuring hardness of tablets, ten tablets were chosen from a series and placed in hardness tester. Then, the hardness was recorded. For the investigation of the thickness uniformity, the thickness of 10 tablets from each series was measured by the caliper and the average was calculated. Dissolution tester (DT 800, Germany) was applied to measure the opening time of the tablet. Single punch (EK-0, Germany) was applied to compress the powder.

*Friability*

For the study of the friability of tablets, 10 tablets were selected from a series and weighed. Then, they were placed in tablet friability tester for 4 min with a rotation speed of 25 rpm. After that, the tablets were fell from a height of 15 cm. Then, the tablets were weighed and their friability percentage was calculated according to the following Equation:

F = (W_1_ – W_2_)/W_1_ × 100 

(Equation 3)

Where W_1_ and W_2_ are the initial and final tablet weights, respectively (20).

*Weight deviation test*

Ten tablets were randomly selected from a series, weighed individually, and the average weights were calculated. Based on the British Pharmacopoeia, the maximum deviation for tablets that their weigh are less than 250 mg and more than 80 mg, is 7.5%. Also, the same amount of deviation is allowed for tablets whose weight is less than 324 mg and greater than 130 mg. Otherwise, the tablets will be rejected because of the lack of uniformity in weight that may indicate a lack of uniformity in the amount of drug in the tablets.

*FT-IR analysis*

Additional information regarding the structure and any interaction between drug, polymer and other tablet excipients were studied using a FT-IR spectrophotometer (Thermo Nicolet NEXUS 670 FT-IR). FT-IR spectrums of test samples were obtained in the range of 4000–400 cm^−1^ at a resolution of 0.5 cm-1, using the KBr pellet technique. The system was operated in transmission mode.

*DSC thermogram*

DSC thermograms of test samples (meloxicam powder and buccal tablet formulation (B2)) were recorded by a differential scanning calorimeter (DSC; METTLER). Test samples (5 mg) were sealed in an aluminum pan and then heated from 30 to 430 ºC at a heating rate of 20 ºC min^-1^ under a constant nitrogen flow rate (30 mL min^-1^). From the obtained thermograms, melting onset and peak of test samples were detected by the STARe SW 9.01 software.

*In-vitro drug release analysis*

This test was performed by the dissolution tester according to USP38 under the following conditions:

Dissolution apparatus 2 – paddle, rate of 50 rpm, temperature of 37 ºC, pH of 6.8, volume of 900 mL, volume of taken sample = 5 mL, sampling intervals = 0.5, 1, 2, 3, 4, 5, 6, 7, 8, 9, 10, 11, and 12 h, and time of experiment = 12 h. From each set of formulations, 3 tablets were randomly selected. In each compartment of the device, one tablet was placed and after rotation, sampling was performed according to the above conditions. After each sampling, the amount of solution was replaced with 5 mL of phosphate buffer (pH 6.8). Then, the absorbance of samples in λ_max_ of meloxicam was recorded by the spectrophotometer and according to the standard equation, the amount of drug released was determined in each sample.

*Swelling index*

Three tablets from each series were chosen randomly, and with a drop of water and a little pressure, each one was pasted to the lamella. Then, distilled water was poured into a petri dish with a volume of 20 mL, and each of the pasted tablets to the lamella after the distribution was placed into the petri dishes. Afterward, the tablets were brought out in 1 to 12 h, and the surrounding water was dried by filter paper and they weighed. In the end, absorption percentage of water in the pills was calculated by the Equation 4.

Swelling index = [(w_2 _- w_1_)/w_1_] × 100 

 (Equation 4)

Where w_2 _and w_1 _are the weight of pill with water and initial weight of pill, respectively (21).

*Determination of mucoadhesive strength*

Mucoadhesive strength was determined by means of a simple apparatus which had two platforms located in a vertical axis, with an adjustable distance from each other. Freshly obtained sheep buccal mucosal membrane (the papillae were all removed) were stored frozen in phosphate buffer pH 6.8 for 1 day. After this period, the mucosal tissue of sheep was thawed to room temperature before use. At the time of testing, the mucoadhesive tablet was fastened to the upper platform by cyanoacrylate glue. Also, a section of the buccal mucosal tissue was fastened to the lower platform. Then, the lower platform that the buccal mucosal tissue bonded on it, placed into the cell containing phosphate buffer. Next step, the mucosal surface was exposed to the mucoadhesive tablet in order to adhere to it. Thereafter, the lower platform was slowly moved down at a rate of 1 mm/min, until the mucoadhesive tablet completely separated from mucosal tissue. The maximum force needed for the separation of the two platforms from each other was measured and reported as the mucoadhesive strength of the tablet. Each test was carried out in thrice, and results were reported as mean ± SD.

*Modification of taste*

Meloxicam is a bitter powder, and in order to increase the compliance of the patients, some excipients were added to the formulations for the taste modification. The final formula was given to 10 volunteers to determine the quality of the taste tablets. For this purpose, formulation B_2_ was chosen and Sucralose, Sodium Chloride, and Citric Acid were used, as sweetener, covering bitter taste, and the more suitable taste, respectively. The components of this formulation are represented in Table 4.

## Results and Discussion

*Standard plot of meloxicam*

**Figure 1:**
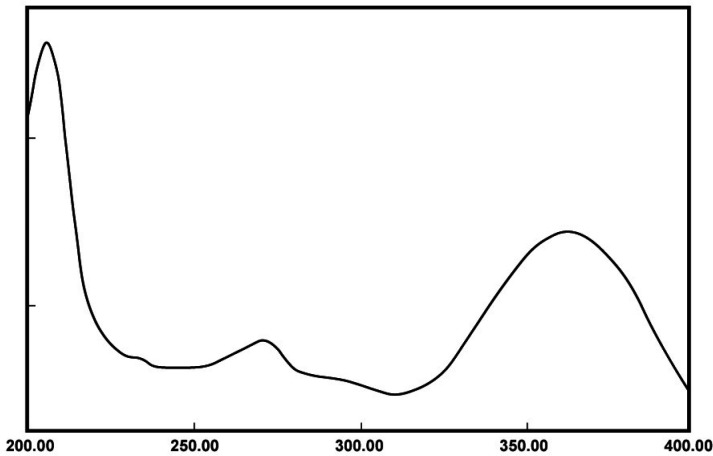
UV spectrum of tested meloxicam powder

The UV spectrum of meloxicam powder in phosphate buffer (pH 6.8) is demonstrated in Figure 1. As shown in Figure 1, the wavelength of maximum absorption for meloxicam is 362 nm, which matches the _max_ reported in previous studies (22).

**Figure 2:**
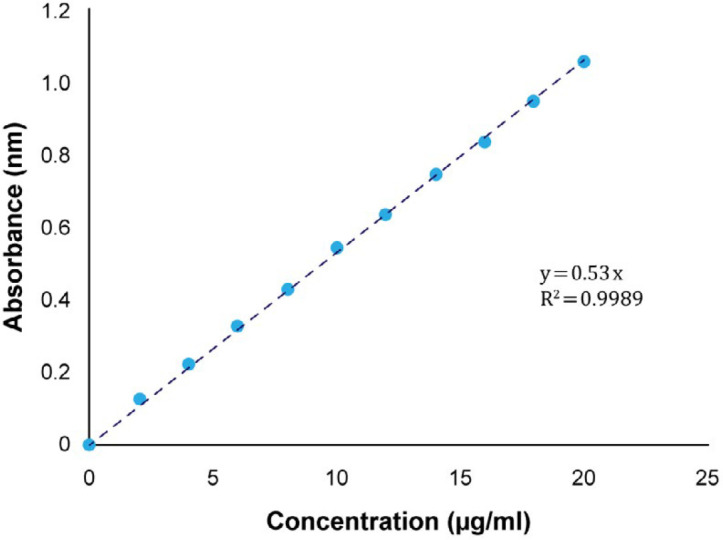
Calibration curve of meloxicam in phosphate buffer (pH 6.8) in wavelength of 362 nm

Using the standard curve, the amount of released drug was determined. Therefore, the absorbance of standard solutions versus their concentration was plotted and the equation was obtained. As shown in Figure 2, the regression coefficient (R^2^) equal to 0.9989 represents the linearity of the calibration curve. Therefore, the use of this curve to determine the amount of released meloxicam at various times is acceptable.

*Results of Carr’s index and Hausner ratio*

The results of using the formulas of apparent density, condensed density, Carr’s index, and Hausner ratio are presented in Table 5 after three times test. To evaluate the powder flow, the obtained results in Table 5 were compared with the reference that including the Carr’s index and the Hausner ratio. Based on Hausner coefficient, if the obtained number is less than 1.25, the powder flow is suitable and if it is more than 1.25, it is undesirable. If the Hausner coefficient is between 1.25 and 1.5, powder flow can be modified by the suitable lubricant (23). Also, the Carr’s index < 16%, and 16–23% indicates a good and medium flow of powder respectively. According to these results, meloxicam powder is not desirable (24). Therefore, it needs to use excipient to solve the problem of flowability of the powder.

*Results of physical control experiments*

The range of hardness for buccal mucoadhesive tablets is 8-12 kPa in the sources, the hardness of all formulations is within the permitted range (25). According to Table 6, there is no constant increasing or decreasing process in formulations A_1_ to A_6_, B_1_ to B_4_, and C_1_ to C_3_, and there is a significant difference between them. Formulations A_5_, B_1_, and C_1_ have the highest hardness and formulations A_4_, B_4_, and C_4_ have the lowest hardness. Also, Table 6 shows that the thickness of the prepared tablets does not have constant increasing or decreasing trend in all formulations A_1_ to C_3_ and there is a significant difference between them. In the sources, the thickness difference of ±5% is acceptable and all of the tablets in this series are in the range (26). In addition, formulations A_4_ and A_6_ have friability percentage more than 1% because of their lesser hardness than other formulations in this series. For tablets weighing more than 80 mg and less than 250 mg, permitted percentage of mean weight deviation is ±7.5. Therefore, all formulations A_1_ to C_3_ are within the permitted range and there is no significant difference between them.

*FT-IR spectrum analysis*

**Figure 3.**
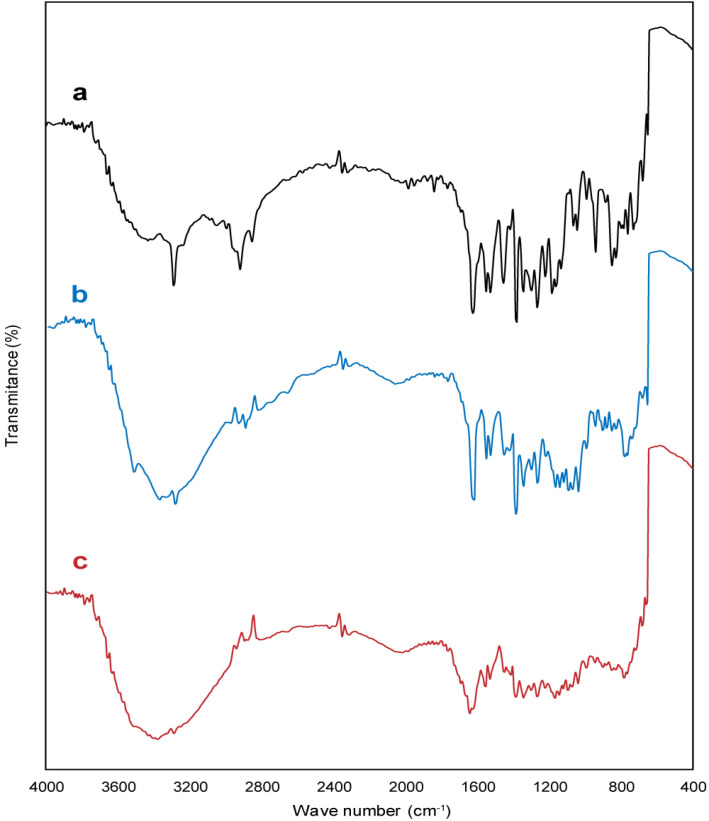
FTIR spectra of (a) pure meloxicam powder, (b) buccal tablet formulation without meloxicam, and (c) buccal tablet formulation (B2)

The characteristic bands of the meloxicam (Figure 3a) are bands at 3289 cm^−1^ (stretching vibration of a amide group (N–H)), 2917 cm^−1^ (stretching vibration of alkyl group), 1622 cm^−1^ (stretching mode of amide group), the sharp band at 1522 cm^−1^ (C=C aromatic stretching vibration), bands at 1384 and 1172 cm^−1^ (for two sulphonyl groups (S=O stretching vibration)) (27). FT-IR spectrum of buccal tablet formulation without meloxicam displayed characteristic bands related to carbopol 934p and the other excipients. Also, the characteristic bands of meloxicam, carbopol 934p, and the other excipients were observed in FT-IR spectrum of buccal tablet formulation (B2). However, a remarkable decrease appeared in the intensity of the bands, especially the bands related to hydroxyl, amide, and carbonyl groups which may be due to the compression process, leading to high physical interactions, such as the formation of hydrogen bonds between hydroxyl, amide and carbonyl groups. Thus, FT-IR spectrum analysis ruled out the existence of any incompatibility between meloxicam, carbopol 934p, and the other excipients buccal tablet formulation (B2).

*DSC analysis*

**Figure 4:**
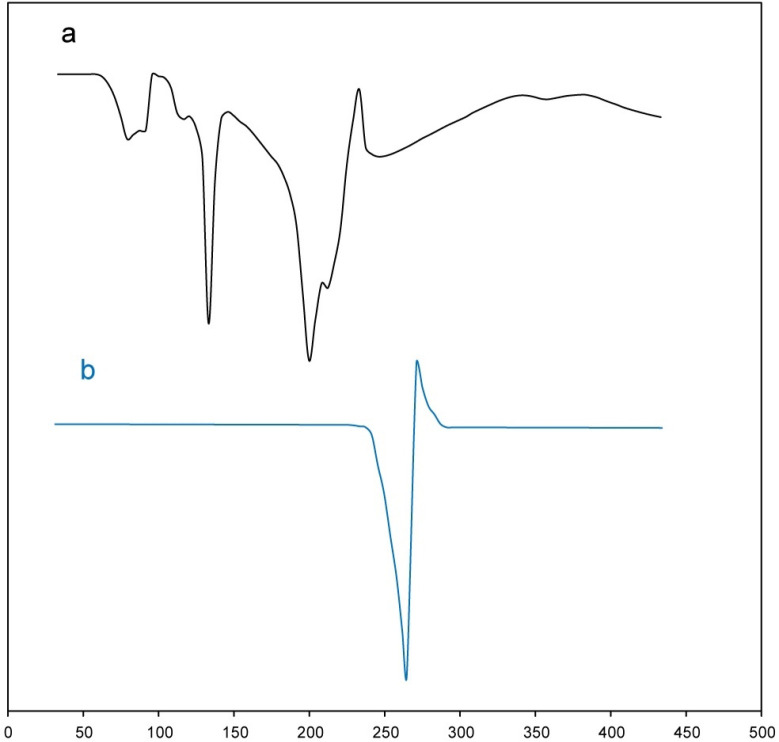
DSC thermograms of (a) buccal tablet formulation (B2), and (b) pure meloxicam powder

DSC thermogram of meloxicam powder and buccal tablet formulation (B2) is shown in Figure 4. As is obvious in Figure 4, DSC thermogram of meloxicam powder showed a melting onset at 248.92 °C and peak at 263.59 °C (28). Melting temperature range of meloxicam does not overlap with the thermograms of the other tablet excipients. Also, DSC thermogram of buccal tablet formulation (B2) showed characteristic peaks of each excipient at the corresponding temperature range. However, for meloxicam, the temperatures related to melting onset and peak in DSC thermogram of buccal tablet formulation (B2) slightly decreased from 248.92 °C and 263.59 °C to 241.43 °C and 253.37 °C, respectively. This phenomenon could be due to the following reasons: first, the existence of high amount of excipients as compared to meloxicam in buccal tablet formulation (B2); second, the drug crystals surrounded by the other tablet excipients getting in contact with meloxicam more closely during the tablet compression. Moreover, compression can lead to breaking in crystal structures of tablet excipients, resulting in a closer contact. Thus, the results obtained from DSC analysis were consistent with FT-IR results, indicating the absence of chemical interactions and the presence of physical interactions between the drug and the other tablet excipients in the buccal tablet formulation (B2).

*In-vitro drug release analysis*

**Figure 5:**
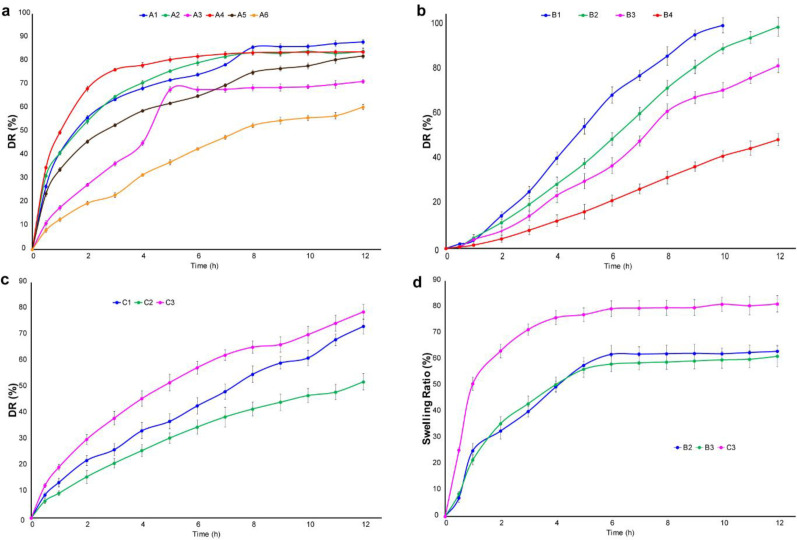
Release of meloxicam from formulations (a) A1 – A6, (b) B1 – B4 and (c) C1 – C3, (d) swelling index of formulations B2, B3, and C3

The tablet prepared in this study is buccal mucoadhesive. Therefore, it should possess the properties of a continuous release system and release the drug over a long period of time. So, the dissolution time for the meloxicam tablet was chosen 12 h. By reviewing the articles, USP38, as well as the monograph of meloxicam, a standard was considered for the release of this drug at specified times (0.5-12). The first formulation of the series A is the formulation A_1_. The results showed that 40% of the drug was released within 1 h, as well as t_50%_ of the drug is less than 2 h. So, it indicates that the release of the drug is rapid and it is not desirable. In Formulation A_2_, with 10 mg of polymer, t _50%_ is approximately 2 h and t _80%_ is 6 h. By increasing the polymer to 20 mg in formulation A_3_, t_50%_ and t_80%_ of the drug reached to 4 h and above 8 h respectively. Significant amount of drug has not been released at this time, which is not desirable. In formulations A_4_, A_5_, and A_6_, with increasing grade of polymer, it is expected that the power of absorption of water will increase, as a result, the pores will be generated in the tablet through which the drug will be released. This happened with formulation A_4_ and t_80%_ reached to 4 h. After that, the release was stopped and a little amount of drug was released. The results showed that in the formulation A6 with 20 mg polymer, t_50%_ reached to 7.5 h, but after 8 h, approximately 52% of the drug was released, which is not suitable. As a result, the weight of 15 mg was selected and its release was investigated in formulation A_5_. It was found that t_50%_ is 2.5 h and after 8 h 75% of the drug has been released. Thus, it has a better release profile than formulations A_4_ and A_6_. The results of the release related to formulation A are shown in Figure 5a. On the other hand, the release of formulation B_1_ containing 10 mg of polymer showed that t_50% _and t_80%_ are 4.5 and 7.5 h respectively. This process is fairly rapid and the drug may be finished at 9 h, so this formulation is not acceptable. Then, the formulation of B_2_ containing 20 mg was studied. It was observed that t_50%_ is approximately 6 h and has not reached 80% after 8 h due to the high weight of the polymer and strong gel structure. As a result, the release was investigated up to 12 h. Also, the formulation B_3_ containing 10 mg of the polymer indicated that t_50%_ was about 7 h, but it did not reach to t_80%_ after 8 h. The process of release was good and the study continued for 12 h. But, 82% of the drug was released after 12 h, which is slow and is not appropriate for this drug. In addition, formulation B_4_ including 20 mg of polymer was used that was very slow release and 32% of the drug was released after 8 h. The results of this formulation are presented in Figure 5b. In the formulation C_1_, it was observed that after 6 h, 50% of the drug and after 8 h, 60% of the drug was released. Hence, the test was surveyed for 12 h. The results showed that 73% of the drug was released and 12% of it was remained after 12 h, which is not desirable. In the formulation C_2_, after 8 h, 41% of the drug was released because the HPMC10000 polymer is more than the other polymers and power for making the gel network is high. So, this formulation is not suitable. Furthermore, the formulation C_3_ showed that after 7 and 8 h, 50% and 55% of the drug released, respectively. So, the experiment was continued for 12 h. After 12 h, 78% of the drug has been released, which is not desirable. The results of this formulation are illustrated in Figure 5c. Among all the formulations that were selected for the 12 h release, it was found that formulation B_2_ with the maximum release of 99% has the most appropriate process. A. R. Gardouh *et al.* reported that the polyvinyl alcohol (2% w/w)-propylene glycol (20% w/w) buccal film has fast meloxicam release profile (about 100% meloxicam released in 3 h), whereas buccal tablets fabricated in the present project possess a sustained release profile for meloxicam (about 100% meloxicam released in 12 h) (29). Therefore, this formulation was used to modify the taste.

*Drug release kinetic analysis*

Zero-order, first-order, and Higuchi kinetics were investigated for each selected formulation. In the zero-order model, the amount of the dissolved drug is plotted against time and the amount of obtained slope indicates the dissolution rate. This kinetic can be expressed by Equation 5.

Q_t_ = Q_0_ + k_0_t (Equation 5)

Where Q_t_, Q_0_, and k_0_ are amount of dissolved drug at time t, initial amount of dissolved drug, and zero order release constant, respectively. Also, the first-order model is used when the graph of the amount of the dissolved drug versus time is not linear. In this model, the logarithm of the amount of dissolved drug is plotted against time and slope of it expresses constant of dissolution rate.

log C = log C_0_ - Kt/2.303 

 (Equation 6)

Where C_0_, K, and t are the initial concentration of drug, the first order rate constant, and the time respectively.

Furthermore, the Higuchi model is used to evaluate the beginning of 80% mechanism of release. According to this model, the amount of the dissolved drug is plotted against the square root of time that slope value indicates the constant of dissolution rate. The equation of this model is defined as follows:

Q = k_H _× t^1/2 ^

 (Equation 7)

Where Q is dissolved drug and k_H_ shows Higuchi dissolution constant (30).

The results of zero-order, first-order, and Higuchi kinetics related to formulations B_2_, B_3_ and C_3 _are shown in Table 7. The kinetic results related to formulations B_2_, B_3_ display that the highest correlation coefficient is related to zero-order kinetic, whereas, the comparison of various kinetics related to the formulation C_3 _represents that the highest R^2^ is related to Higuchi kinetic.

*Results of swelling test*

The selected three formulations (B_2_, B_3_, and C3) were reviewed. The plot of swelling index versus time is demonstrated in Figure 5d. Among them, the swelling rate of formulation C_3_ is very high and has been observed the rapid process in the release test. Formulation B_2_ reached its maximum swell rate at 5 h and absorbed water is 57.76% of its weight. Also, swelling profile of formulation B_3_ is similar to that of formulation B_2_.

*Results of the mucoadhesive strength analysis*

The results obtained from the mucoadhesive strength analysis of the selected three formulations (B2, B3 and C3) are given in Table 8. The results presented in Table 8 showed that the polymer type affected their mucoadhesive strength. Also, they demonstrated that B2 formulation had the highest mucoadhesive strength. A possible description for this might be that polymer used in this formulation is carbopol 934p, and this polymer is a member of poly (acrylic acid) family, which can remarkably interact with mucosa by hydrogen bonding. Therefore, formulation B2 possessed excellent mucoadhesive strength.

*Analysis of the amount of active ingredient in the selected formulations*

The calibration curve of Figure 2 has been used to determine the amount of active ingredient. As shown in Table 9, the calculated values of the active ingredient are within the permitted and accepted range of pharmacopeia (90-110%).

*The results of modifying the taste*

Primary formulations of taste modification were given to 4 volunteers and 3 of them were selected. Then, three acceptable formulations (D_7_, D_8_, and D9) were given to 10 healthy and non-smokers volunteer (between age 20-27) and announced a number between 1 – 6. Finally, the D_8_ formulation was selected to eliminate and improve the bitter taste of selected tablet. The results are summarized in Table 10.

**Table 1:** The components of single formulation using cellulose polymers

	**Formulation**					
**Weight (mg)**	**A**_1_	**A**_2_	**A**_3_	**A**_4_	**A**_5_	**A**_6_
Meloxicam Avicel PH101 Lactose Magnesium stearate Aerosil HPMC 1000 HPMC 4000 HMPC 10000 Total weight of tablet	15 12 40 2 1 30 --- --- 100	15 12 60 2 1 --- 10 --- 100	15 12 50 2 1 --- 20 --- 100	15 12 60 2 1 --- --- 10 100	15 12 55 2 1 --- --- 15 100	15 12 50 2 1 --- --- 20 100

**Table 2:** The components of single formulation using carbopol

	**Formulation**			
**Weight (mg)**	**B**_1_	**B**_2_	**B**_3_	**B**_4_
Meloxicam Avicel PH101 Lactose Magnesium stearate Aerosil Carbopol 934p Carbopol 971p Total weight of tablet	15 12 60 2 1 10 --- 100	15 12 50 2 1 20 --- 100	15 12 60 2 1 --- 20 100	15 12 50 2 1 --- 20 100

**Table 3.** The components of the combined formulation using cellulose derivatives

	**Formulation**		
**Weight (mg)**	**C**_1_	**C**_2_	**C**_3_
Meloxicam Avicel PH101 Lactose Magnesium stearate Aerosil HPMC 4000 HPMC 10000 Total weight of tablet	15 12 50 2 1 10 10 100	15 12 50 2 1 7 13 100	15 12 50 2 1 13 7 100

**Table 4:** The components of formulation for modification taste

	**Components (mg)**		
**Formulations**	**Sucralose**	**Sodium chloride**	**Citric acid**
D_1_ D_2_ D_3_ D_4_ D_5_ D_6_ D_7_ D_8_ D_9_	5 5 3 3 3 3.5 2 2 2	7 5 10 7.5 5 7.5 7.5 7.5 7.5	5 5 5 5 5 5 5 7.5 5

**Table 5:** Results of Carr index and Hausner ratio

	**Sample**			
**Parameters**	**1**	**2**	**3**	**Average**
Bulk density (g. cm^3^) Dense density (g. cm^3^) Carr index Hausner ratio	0.56 0.86 34.88 1.54	0.54 0.89 39.33 1.65	0.57 0.96 40.63 1.68	55.67±1.25 90.33±4.19 38.28±2.46 1.62±0.06

**Table 6:** Results of quality control test on different formulations.

	**Parameters**			
**Formulation**	**Weight (mg) n = 20**	**Thickness (mm) n = 10**	**Hardness (kgf) n = 10**	**Friability (%) n = 1**
A_1_ A_2_ A_3_ A_4_ A_5_ A_6_ B_1_ B_2_ B_3_ B_4_ C_1_ C_2_ C_3_	101.2±2 100.1±2.1 99.1±4.1 100.4±3.44 98.9 ±1.9 97.9±3.9 97.6±1.2 99.0±0.4 98.8 ±1.68 101.2 ±1.68 98.2±1.23 99.8 ±1.63 101.9 ±2.84	2.55±0.07 2.62±0.06 2.26±0.05 2.27±0.03 2.12±0.02 2.2±0.02 2.3±0.01 2.7±0.11 2.85±0.05 3.3±0.48 1.92±0.02 2.32±0.01 2.87±0.04	7.8±0.51 7.4 ±0.21 7.1±0.25 6.2±0.1 7.9±0.34 6.9±0.24 8.1±0.3 7.7 ±0.2 7.4±0.45 6.8±0.6 8.3±0.68 7.5±0.28 7.8±0.27	0.86±0.06 0.81±0.03 0.89±0.02 1.6 3±0.02 0.52±0.04 1.39±0.05 0.31±0.02 0.42±0.01 0.68±0.08 0.57±0.03 0.46±0.02 0.61±0.07 0.65±0.03

**Table 7:** Kinetic model parameters for test samples; R^2^: regression coefficient

**variables**	**Test samples**		
	**B**_2_	**B**_3_	**C**_3_
$R_{0}^{2}$	0.99	0.98	0.70
$R_{1}^{2}$	0.36	0.56	0.48
$R_{H}^{2}$	0.79	0.78	0.99

**Table 8:** The content of the mucoadhesive strength in the selected formulations (n = 3).

**Formulation**	**Content of the mucoadhesive strength (g), g** ± **SD**
B_2_ B_3_ C_3_	35. 3 ±2.1 20.7±1.8 15.4±1.1

**Table 9:** The amount of active ingredient in the selected formulations (n = 3).

**Formulation**	**Amount of active ingredient (mg), mg** ± **SD**
B_2_ B_3_ C_3_	15.523 ±2.17 16.664±1.97 14.012±1.92

**Table 10:** Results of taste modification on B_2_ formulation

**Formulation**	**No. of volunteer**									
	**1**	**2**	**3**	**4**	**5**	**6**	**7**	**8**	**9**	**10**
D_7_ D_8_ D_9_	5 6 5	4 6 5	4 6 5	4 6 4	5 5 5	3 4 4	4 6 5	4 6 4	4 5 4	5 6 5

## Conclusion

In the present study, buccal mucoadhesive tablet of meloxicam was successfully prepared. Among the 13 formulations, the formulation B_2_ using HPMC polymer had suitable swelling ratio and drug release profile. Also, the formulation B_2_ followed zero order kinetics. Thus, the meloxicam mucoadhesive buccal tablet can be a suitable choice to bypass the extensive hepatic first-pass metabolism with a betterment in the bioavailability of meloxicam via buccal mucosa. Finally, the superior formulation (formulation B2) can be used for large-scale production by direct compression.
